# Milk Formula Diet Alters Bacterial and Host Protein Profile in Comparison to Human Milk Diet in Neonatal Piglet Model

**DOI:** 10.3390/nu13113718

**Published:** 2021-10-22

**Authors:** Fernanda Rosa, Boris L. Zybailov, Galina V. Glazko, Yasir Rahmatallah, Stephanie Byrum, Samuel G. Mackintosh, Anne K. Bowlin, Laxmi Yeruva

**Affiliations:** 1 USDA-ARS, Arkansas Children’s Nutrition Center, Little Rock, AR 72202, USA; ftrindadedarosa@uams.edu; 2 Department of Pediatrics, University of Arkansas for Medical Sciences, Little Rock, AR 72201, USA; 3 Department of Biochemistry and Molecular Biology, University of Arkansas for Medical Sciences, Little Rock, AR 72201, USA; BLZybaylov@uams.edu (B.L.Z.); sbyrum@uams.edu (S.B.); MackintoshSamuelG@uams.edu (S.G.M.); 4 Department of Biomedical Informatics, University of Arkansas for Medical Sciences, Little Rock, AR 72201, USA; GVGlazko@uams.edu (G.V.G.); YRahmatallah@uams.edu (Y.R.); 5 Department of Microbiology and Immunology, University of Arkansas for Medical Sciences, Little Rock, AR 72201, USA; Abowlin@uams.edu

**Keywords:** neonates, intestinal, microbiota, metaproteome

## Abstract

The metaproteome profiling of cecal contents collected from neonatal piglets fed pasteurized human milk (HM) or a dairy-based infant formula (MF) from postnatal day (PND) 2 to 21 were assessed. At PND 21, a subset of piglets from each group (*n* = 11/group) were euthanized, and cecal contents were collected for further metaproteome analysis. Cecal microbiota composition showed predominantly more Firmicutes phyla and *Lachnospiraceae* family in the lumen of cecum of HM-fed piglets in comparison to the MF-fed group. *Ruminococcus* *gnavus* was the most abundant species from the Firmicutes phyla in the cecal contents of the HM-fed piglets at 21 days of age. A greater number of expressed proteins were identified in the cecal contents of the HM-fed piglets relative to the MF-fed piglets. Greater abundances of proteins potentially expressed by *Bacteroides* spp. such as glycoside enzymes were noted in the cecal lumen of HM-fed piglets relative to the MF. Additionally, lyases associated with *Lachnospiraceae* family were abundant in the cecum of the HM group relative to the MF group. Overall, our findings indicate that neonatal diet impacts the gut bacterial taxa and microbial proteins prior to weaning. The metaproteomics data were deposited into PRIDE, PXD025432 and 10.6019/PXD025432.

## 1. Introduction

Studies have demonstrated positive health outcomes in human milk fed in comparison to formula-fed infants. There is considerable amount of evidence showing that human milk diet minimizes risk of necrotizing enterocolitis in preterm infants [1,2,3,4]. Additionally, the gut microbiota colonization can be influenced by neonatal diet. The literature suggests that breastfed infants have higher abundance of Bifidobacteria and Bacteroides than formula-fed infants [5,6,7,8,9,10]. Previously, we have reported that human milk-fed (HM) piglets had higher fecal Bacteroides abundance relative to a formula-fed group (MF), as well as a stronger immune response by which enhanced T cell proliferation in the mesenteric lymph nodes of HM-fed animals [11]. Gut and immune health are functions of both diet and gut microbes that respond to diet. Several metabolites are known to derive from the microbial metabolism throughout the intestinal regions [12,13]. For instance, indigestible carbohydrates can be fermented by distal gut bacteria (cecum and colon) to short-chain fatty acids [14], and complex human milk oligosaccharides (HMOs) are broken down by microbes in the distal gut, serving as substrates to commensal bacteria among other functions reviewed elsewhere [15]. In addition, derivatives of the tryptophan metabolism (i.e., indoles) [16] and the conversion of primary to secondary bile acids are also metabolized by distal gut microbiota [17]. Metabolomics analysis of the large intestinal contents of these piglets revealed that HM feeding resulted in greater abundance of fatty acids, polyamine derivatives, glutamic acid, and tryptophan metabolites in the distal gut of HM-fed piglets. In contrast, MF-fed piglets had greater abundance of cholesterol, bile acids, and amino acids in the distal colon at 21 days of age relative to the HM-fed group [18]. These findings might be a result from the interaction between neonatal diets and gut microbial activity.

Several approaches have demonstrated that microbiota compositional changes can be altered in response to diet [19,20,21,22,23,24,25,26,27]. However, studies were limited in terms of determining the functional relevance of the microbial changes and which components of microbiota play a role in positive health outcomes observed in human milk-fed infants. Newer technology such as metaproteomics might help to determine the microbial protein presence, abundance, and microbial community. This allows us to understand the functional role of microbiota and their interactions with host and other microbial species in an ecosystem. In addition, host proteins can be identified from the sloughed off cells of the gastrointestinal tract. The proteins provide a measure of the activity of the cells and their abundances provide a phenotype at the molecular level. Metaproteomics was used often to study environmental samples in the 2000s [28]. The first shot gun metaproteomics from human samples was conducted by Verberkmoes et al. in 2009 [29]. They identified that 30% of protein hits were associated with the host, and several microbial pathways related to carbohydrate and energy metabolism were observed. The literature is limited in terms of its understanding of the microbial protein/peptide functions, especially in neonates.

We hypothesized that cecal bacterial community and bacterial proteins act as signaling molecules to promote gut homeostasis and immune function in HM-fed piglets relative to MF-fed piglets. Thus, a metaproteomics approach was used to determine the bacterial protein expression in piglets fed either human milk or cow’s milk formula.

## 2. Materials and Methods

### 2.1. Experimental Design

An animal experiment was conducted as per the Institutional Animal Care and Use Committee approval at the University of Arkansas for Medical Sciences (UAMS Institutional Animal Care and committee 3727 and 3471). Diet composition and the experimental design were published previously [11,30]. Piglets were obtained from 4–6 sows. At 2 days of age, White Dutch Landrace Duroc male piglets were randomized into two dietary groups (*n* = 11/group): pasteurized human milk (HM) provided from the Mother’s Milk Bank of North Texas, or a dairy-based infant formula (MF) (milk formula; Similac Advance powder; Ross products, Abbott Laboratories, Columbus, OH). Piglets were fed to meet the nutrient requirements of growing pigs as per National Research Council (NRC) guidelines [31]. Piglets were euthanized at PND 21 to collect cecal contents. Samples were immediately frozen in liquid nitrogen and transferred to an −80 °C freezer.

### 2.2. Metaproteome

For the comparison of the MF-fed vs. HM-fed piglets, we employed a mass spectrometry-based metaproteomics pipe-line, which was described previously [32,33]. Briefly, steps included (1) protein isolation from cecum samples, (2) fractionation of proteins on SDS-PAGE, (3) in-gel digestion by trypsin, (4) high-resolution mass spectrometry, (5) de novo sequencing, (6) protein inference, (7) organism inference, and (8) differential abundance analyses. To identify peptides and infer proteins, we used PEAKS v.8 (Bioinformatics Solutions, Waterloo, ON, Canada) with the following settings: merge scans—left unchecked; correct precursor—mass only; filter scans—unchecked. The following parameters were used for the de novo sequencing: parent mass error tolerance—5 ppm; fragment mass error tolerance—0.5 Da; enzyme—trypsin; fixed modifications—carbamidomethylation (C); variable modifications—oxidation (M), deamidation (NQ); max variable PTM per peptide—3; report # peptides—5.

Preliminary taxonomy analysis was conducted to evaluate reproducibility of protein extraction. De novo peptide tags obtained by PEAKS for the raw MSMS spectra were filtered using the average local confidence score (ALC ≥ 80), and the filtered peptide lists were supplied to the online metaproteomics tool Unipept [34], with the following settings: equate I and L—checked, filter duplicate peptides—unchecked, advanced missed cleavage handling—checked. The taxonomy information was visualized using a tree diagram provided by Unipept. Bacterial-to-host ratios were calculated using number of de novo sequences matched to each of the two taxonomic kingdoms (Table S1). This step is required the assessment of the quality of protein extraction in order to ensure that sufficient number of bacterial proteins was extracted. After this quality control step, we proceed with the protein identification using the multi-step database strategy as implemented in Peaks Studio.

Multi-step database search strategy for protein identification: The high quality de novo tags (average local confidence score ≥ 50%) were search against a series of protein databases using the multi-step database strategy. The false discovery rate estimation as implemented in PEAKS is compatible with the multi-step searches [35]. Step 1: Uniprot/Tremble protein database (downloaded on 13 April 2020) was searched using *Homo sapiens* and *Sus scrofa* taxonomic filter (310,501 entries were searched). Unmatched de novo tags from this step were passed on to Step 2, wherein the Uniprot database was searched using bacteria, archaea, and fungi as taxonomic filters (142,741,860 entries searched). No filters were applied to the search results in these 2 first steps, apart from the de novo quality score (ALC ≥ 50%). All of the identified entries from the first two steps (≈10% estimated F.D.R at this point, 0 unique peptides allowed) were used to compile a sequence database for the final search. Step 3: The de novo tags were re-searched against the final sequence database derived from the results of the previous two steps (172,464 entries), applying stringent FDR criteria to the final result: 1% false discovery rate for peptide-to-spectrum matches (corresponding average −10lg*P* ≈ 25 across samples) and minimum of 1 unique peptide per protein. One unique peptide hits were further required to have −10lg*P* = 30 in order to be considered identified. Additional filters were applied at the next step for comparative analysis.

Differential abundance of proteins and bacteria: Spectral counts (number of tandem MS spectra that match to a given protein sequence via the database search) were used to infer differential abundant (DA) proteins and taxonomic units. At the taxonomic unit level, the spectral counts of proteins were grouped using taxonomic information in the sequence database and then were summed to obtain total spectral counts for each species in each sample. If species were not identifiable, higher taxonomic levels were used. Moreover, the identified organism had to be present in at least 4 of the independent biological replicates in either of the two conditions compared. The counts were filtered so that species with less than 10 counts in all samples, but one was removed. Then, counts were normalized to the trimmed mean of M values, a method frequently employed in RNA–Seq analysis [36]. The differential abundance analysis was performed employing Poisson–Tweedie family of distributions using tweeDE package in R [37]. Initially, data analysis for microbiota and microbial and host proteins was conducted by edgeR and DESeq2 methods with different statistical tests (i.e., Wald LRT for DESeq2 and LRT, exactTest for edgeR). Finally, Benjamini–Hochberg correction was used for multiple testing to define differentially abundant proteins and bacterial species (FDR < 0.05).

### 2.3. Data Accessibility

The mass spectrometry proteomics data were deposited to the ProteomeXchange Consortium via the PRIDE [38] partner repository with the dataset identifier PXD025432 and 10.6019/PXD025432. Reviewer login details: Username: reviewer_pxd025432@ebi.ac.uk; password: qvFTwXRs.

## 3. Results

**Figure 1:**
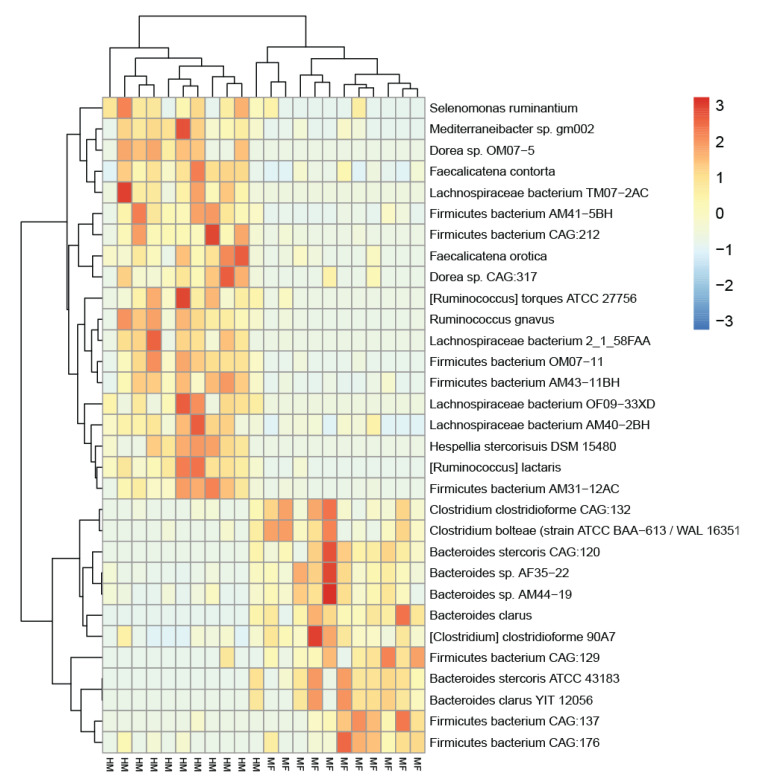
Metaproteomics revealed distinct microbiome profiles in human milk-fed and milk formula-fed neonatal piglets. Heatmap visualization of the top 31 significantly abundant bacteria is shown. The two phenotypes are indicated by the bottom labels: HM and MF (*n* = 11/group). Unsupervised clustering of the metaproteomics-derived bacterial abundance resulted in HM and MF piglets being grouped together, except for one outlier from the HM group.

**Figure 2:**
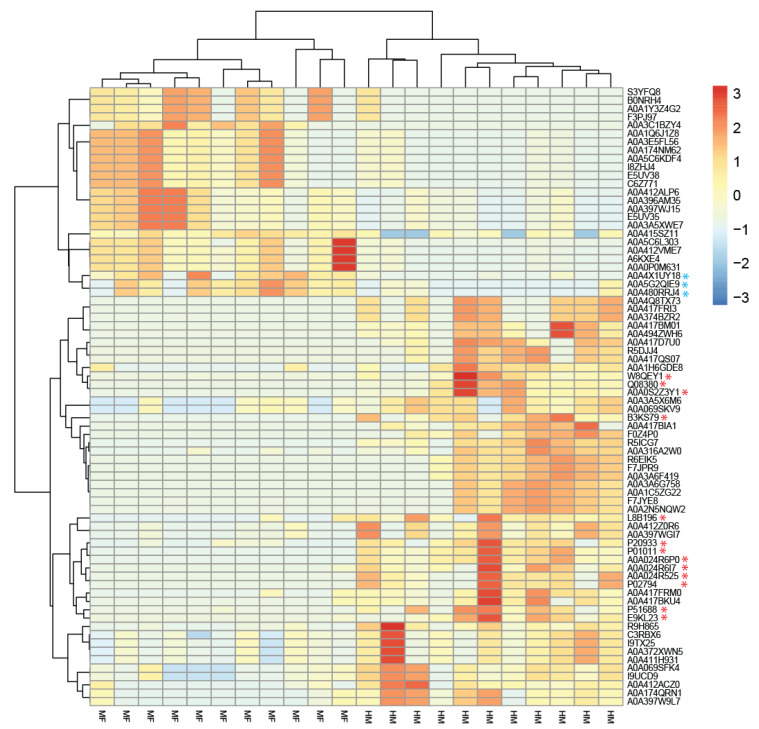
Metaproteomics-derived protein profiles uniquely defined in HM and MF-fed neonatal piglets. Heatmap constructed from differentially abundant proteins is shown. The two dietary groups are indicated by the bottom labels: HM and MF (*n* = 11/group). Unsupervised clustering of bacterial and host protein resulted in HM and MF piglets being grouped together. Host and human proteins are identified with *, red—human, blue—pig.

Figures S1 and S2 detail these data as Venn diagrams (Bacteria Venn pairwise and protein Venn pairwise, respectively). Due to zero inflation and overdispersion observed with the data, we used the Poisson–Tweedie method, which enables direct fitting of data with heavy-tails and/or zero-inflation [32]. Heat maps show the most abundant bacterial taxa and proteins altered in cecal lumen due to neonatal feeding (Figure 1 and Figure 2, respectively). The microbial abundance and metaproteome data are listed in Table 1 and Table 2. All the bacteria and proteins identified in this study are presented in the Supplemental Datasets S1 and S2, respectively.

### 3.1. Microbial Taxonomy Identification in the Cecal Lumen

The bacterial abundance in the luminal cecum of HM- and MF-fed piglets is shown in Table 1. The cecum profile of the HM-fed piglets was predominantly composed of the Firmicutes phylum and of the *Lachnospiraceae* family, including the species *Ruminococcus lactaris*, *Ruminococcus gnavus*, and *Lachnospiraceae bacterium*, while the cecal lumen of the MF-fed relative to HM-fed piglets had higher abundance of the Bacteroides genera including *Bacteroides clarus* and *Bacteroides stercoris*. Additionally, the cecum of MF-fed piglets had greater abundance of the *Clostridium clostridioforme* (fold-change (FC) = 2.9) compared to the HM-fed group.

### 3.2. Bacterial Proteins Impacted by Diet Groups in the Lumen of Cecum at PND 21

Bacterial peptide profile of cecal contents of HM- or MF-fed piglets at PND 21 are shown in Table 2. A greater number of bacterial proteins were identified in the HM-fed group relative to the MF piglets. The top 10 bacterial proteins identified in the lumen of cecum of MF group were from the phylum Bacteroidetes, including species from Bacteroides and Phocaeicola genus. Peptides derived from *Phocaeicola vulgatus* (*Bacteroides vulgatus*) included RagB/SusD family nutrient uptake outer membrane proteins as well as malate dehydrogenase. In fact, proteins associated with *Phocaeicola vulgatus* were also identified in the cecal contents of the HM-fed piglets; however, a greater diverse pool of peptides were observed relative to the MF group. For instance, galactose oxidase, sialidase, tetracycline resistance protein, and chaperonin were peptides associated with *Phocaeicola vulgatus* that had higher abundance in the cecum of the HM group compared to the MF group. Additionally, the Lacl family transcriptional regulator associated with the *Firmicutes bacterium* was greater in the cecal lumen of HM (FC = 3) relative to the MF group. L-fucose isomerase, D-ribose pyranase, and chaperonin *Firmicutes bacterium* associated-proteins were greater in the cecal contents of HM compared to MF-fed piglets. The aldehyde-lyase fructose-1,6-bisphosphate aldolase had greater abundance in the cecum of the HM group relative to the MF group. Additionally, this enzyme was associated with different species in the cecum of HM group such as *Lachnospiraceae bacterium*, *Ruminococcus gnavus*, and uncultured *Ruminococcus* sp. The abundance of phosphotransferase acetate kinase was also greater in the cecal contents of HM group, and it was associated with both species *Lachnospiraceae bacterium* and *Clostridium* sp. D5.

### 3.3. Host Proteins Identified in the Cecal Contents at PND 21

Host proteins expressed in the cecal contents of HM-fed versus MF-fed piglets at PND 21 is shown in Table S2. Briefly, the human proteins N-sulphoglucosamine sulphohydrolase, epididymis secretory sperm binding protein, alpha-1-antitrypsin, and lactotransferrin were greater (FC > 5) in the cecum of HM-fed piglets compared to the MF group. In contrast, the MF-fed piglets had greater porcine proteins such as secreted folate binding protein, folate_rec domain-containing protein, and transthyretin relative to the HM-fed group.

## 4. Discussion

This study used a porcine model due to the similarities in the anatomy and physiology of the digestive tract between pigs and humans [39,40]. Previous studies found that different protein sources such as bovine milk, hydrolyzed bovine milk, and soybean formula did not change intestinal trypsin and chymotrypsin and the absorption of nitrogen in the small and large intestine in 3-week-old piglets, similar to the human infants [39]. Furthermore, it has been demonstrated that 3-week-old piglets are suitable for studying parameters of digestion and absorption relative to 3-month-old infants [40]. In our previous study, we observed that MF-fed piglets had an increased microbial diversity and richness across the luminal regions compared to the HM-fed group [26], which is in agreement with microbiota composition findings in infants that have shown higher microbial richness in formula-fed infants [41,42]. Thus, the gut related outcomes from the current study have the potential to be translated to infants consuming human milk or formula.

Metaproteome analysis of gut microbiota are typically conducted with fecal samples, and the latter constitutes a significant amount of microbial biomass in feces, which can reflect the intestinal conditions. However fecal samples are a mixture of microbiota from all intestinal regions, and the piglet model provided the opportunity to measure the specific bioregion of the gut (i.e., cecal contents). In addition, it has been demonstrated that the main microbial fermentation of both carbohydrate and protein occur in the cecum, suggesting a microbiota role in putrefaction [43]; thus, cecal luminal contents were considered for this study. Future studies are needed to determine bioregional differences in bacterial protein expression and its impact on gut health.

Bifidobacterium and Bacteroides are the most abundant genera observed in breastfed infants [24,44], while in formula-fed infants, Bifidobacterium and Bacteroides have been identified in similar levels [9]. *Bacteroides vulgatus* had persistent abundance from birth up to 4 months of age in the infant gut [45]. *Bacteroides vulgatus* and *Bacteroides dorei* abundances have been reported to increase in the feces of infants at 6 months of age [46], while in the adult gut microbiota community of healthy individuals, these species within the Bacteroides genera are the most predominant [47]. Additionally, Bacteroides abundance in the human gut has been associated with the maintenance of a healthy gut [48]. In line with these observations, we previously reported a higher abundance of Bacteroides in the feces of HM-fed piglets relative to the formula-fed group [11]. In the current study, metaproteomic analysis revealed greater abundance of specific bacterial peptides belonging to the *Bacteroides vulgatus* in the cecal contents of HM-fed piglets relative to MF-fed group at 21 days of age. Interestingly, studies have shown that *Bacteroides vulgatus* can grow in the presence of human milk oligosaccharides (HMO), as well as metabolize these complex carbohydrates [49,50]. Moreover, proteins associated with *Bacteroides vulgatus* has been identified in stool samples of breastfed infants at 2–3 months of age [51]. Interestingly, *Bacteroides* spp. promote Treg cell development [52,53], and it has been shown that infants with decreased allergic colitis had increased *Bacteroides* spp. in their stool [54] suggesting the role of these species in promoting immune responses and homeostasis in the gut. This further suggests the role of *Bacteroides* spp. in cell-mediated immunity, but it is yet to be determined how this impacts antibody and humoral immune response.

Recently, Bifidobacterium abundance has been reported to decrease in the feces of infants from 6 to 12 months of age, while *Lachnospiraceae* abundance increased [46]. Interestingly, in this study, alongside the *Bacteroides vulgatus*-associated proteins, a greater number of enzymes related to the *Lachnospiraceae* family were identified in the luminal cecum of HM-fed piglets relative to the MF group. Studies demonstrated that bacteria within the *Lachnospiraceae* family, in particular *Ruminococcus gnavus*, has the ability to produce iso-bile acids, and such metabolites can favor the growth of Bacteroides [55,56]. Recently, the PreventADALL cohort study evaluated the microbial composition and the metaproteome of 100 mother–child pairs from Norway and Sweden [57]. Within the Firmicutes phyla, the predominant species identified in the feces of 12 months old infants was *Ruminococcus gnavus*, and glycoside hydrolases were the enzymes associated with such species [57]. These findings are in agreement with the greater abundance of enzymes involved in the degradation of sialic acids observed in our study, including fructose-1,6-bisphosphate aldolase potentially expressed by *Ruminococcus gnavus* in the luminal cecum of HM-fed piglets relative to MF group at PND 21. *Ruminococcus gnavus* and such aldolase enzymes have been reported to metabolize sialic acid to N-acetylmannosamine [58]. Indeed, sialic acids are a family of carbon sugar acids present in human milk in a rich source of oligosaccharide-bound sialic acid [59].

Furthermore, human milk feeding enhanced the expression of glycoside hydrolases, nutrient uptake proteins, and transporters, which are common enzymes involved in the HMO consumption by *Bacteroides* and *Bifidobacteria* [60,61]. Therefore, it is plausible to acknowledge that milk glycans such as HMOs promote the growth of beneficial bacteria in HM-fed piglets. Additionally, the different hydrolases identified in the HM-fed piglets potentially expressed by *Bacteroides* might benefit the immune system, pending mechanistic data.

We acknowledge that in the current study, numbers of microbial taxa and proteins identified were smaller than in usual metagenome analysis. Kleiner et al. described very elegantly the limitations of metaproteomics data acquisition with current mass spectra and how that limits in-depth community analysis [62]. In addition, the metaproteomics analysis requires a well-curated sequence database to assign the proteins to individual microbial species. This potential mismatch between identified proteins and their assignment to bacterial species is evident when one compares hierarchical clustering using bacterial abundance (Figure 1) and protein abundance (Figure 2). The clustering using protein abundance shows clear separation between the HM and MF groups, while clustering using bacterial abundance has one outlier from the HM group (the right-most HM column in the middle of the heatmap, Figure 1). We argue that the limited number of microbial species and hits to microbial proteins in the current study is a result of combination of technology and data acquisition. This is expected to improve in the next few years with better mass spectra technology and metaproteome database tools.

The current study was conducted at controlled environmental (housed at the vivarium) and isocaloric diets for both HM and MF groups. The human milk used in this study was composed of milk samples ranging from 2 to 12 months of lactation, and different components were added to the diet to maintain the growing piglet nutrient requirements. Additionally, the piglets were enrolled in the study at 2 days of age. Thus, this study lacks data on the colostrum intake. These limitations might introduce variation in the milk composition and might affect the luminal microbiota composition and protein expression of the gut microbiota. Moreover, piglets were from 4–6 sows, by which genetic differences could cause some variation.

## 5. Conclusions

In summary, we observed a 5.4-fold increase in the relative abundance of *Ruminococcus gnavus* in the cecal microbiota composition of HM-fed piglets relative to the MF-fed piglets at 21 days of age. This bacterial abundance was also associated with the expression of glycolytic enzymes in the cecum lumen of HM-fed piglets compared to the MF group. Furthermore, the greater number of proteins potentially expressed by *Bacteroides vulgatus* observed in the cecal contents of HM-fed piglets relative to the MF-fed group at 21 days of age might be associated with the ingestion of bioactive components of human milk (i.e., HMOs metabolized by this gut bacteria) and possibly promotes immune function. Overall, our findings highlight the association between gut microbiota composition upon different neonatal diets with the peptides and enzymes originated from this interaction. We believe that further research in the field of metaproteomics might be crucial to understanding the establishment of key gut colonizers and the overall effect on the host metabolism and immune system.

## Figures and Tables

**Table 1:** Cecal bacterial profile of piglets (*n* = 11/group) fed either with human milk (HM) or milk formula (MF) at PND 21.

Bacterial Organism ^1^	HM ^2^	SD	MF ^2^	SD	log_2_ FC ^3^	*p*-Value ^4^
*Bacteroides clarus*	6.9	27.1	95.8	68.4	−3.8	<0.0001
*Bacteroides stercoris* CAG:120	3.4	13.3	44.5	33.9	−3.7	0.0028
*Bacteroides* sp. AF35-22	39.2	49.2	266.5	194.2	−2.8	0.0076
*Clostridium clostridioforme* CAG:132	3.0	6.2	21.7	21.7	−2.9	0.0076
*Bacteroides stercoris* ATCC 43183	4.0	15.7	35.9	30.4	−3.2	0.0112
*Bacteroides* sp. AM44-19	57.6	62.1	312.0	229.9	−2.4	0.0131
*Firmicutes bacterium* CAG:129	1.8	6.9	18.0	12.8	−3.3	0.0159
*Firmicutes bacterium* CAG:137	2.5	11.2	78.9	82.5	−5.0	0.0164
*Firmicutes bacterium* CAG:176	2.2	3.8	17.0	16.0	−3.0	0.0197
*Bacteroides clarus* YIT 12056	3.4	13.3	32.0	30.7	−3.2	0.0375
(*Clostridium*) *clostridioforme* 90A7	6.7	7.9	26.9	27.2	−2.0	0.0375
*Clostridium bolteae* (strain ATCC BAA-613/WAL 16351)	2.5	6.4	16.2	15.0	−2.7	0.0375
(*Ruminococcus*) *lactaris*	135.1	113.0	3.6	10.9	5.2	<0.0001
*Firmicutes bacterium* OM07-11	82.1	51.3	2.5	5.4	5.1	0.0001
*Ruminococcus gnavus*	303.5	202.5	7.1	15.4	5.4	0.0001
*Firmicutes bacterium* AM41-5BH	199.0	119.8	15.6	15.9	3.7	0.0001
*Mediterraneibacter* sp. gm002	47.7	39.0	4.5	7.7	3.4	0.0009
*Firmicutes bacterium* AM43-11BH	275.4	158.2	19.5	18.6	3.8	0.0012
(*Ruminococcus*) *torques* ATCC 27756	73.2	67.4	3.2	11.2	4.5	0.0028
*Firmicutes bacterium* AM31-12AC	209.2	169.6	6.4	10.8	5.0	0.0067
*Firmicutes bacterium* CAG:212	116.8	57.1	3.5	8.3	5.0	0.0087
*Faecalicatena orotica*	50.3	38.1	6.2	12.6	3.0	0.0131
*Selenomonas ruminantium*	233.3	140.5	41.9	106.8	2.5	0.0152
*Dorea* sp. CAG:317	13.1	11.0	2.0	5.1	2.7	0.0485
*Hespellia stercorisuis* DSM 15480	12.8	11.7	0.0	0.0	NA	0.0152
*Lachnospiraceae bacterium* AM40-2BH	139.2	116.6	33.0	51.5	2.1	0.0152
*Faecalicatena contorta*	164.5	122.5	33.1	41.5	2.3	0.0174
*Lachnospiraceae bacterium* 2_1_58FAA	88.4	73.1	0.3	0.9	8.3	0.0197
*Lachnospiraceae bacterium* TM07-2AC	117.5	112.5	0.0	0.0	NA	0.0197
*Dorea* sp. OM07-5	44.0	36.5	0.0	0.0	NA	0.0389
*Lachnospiraceae bacterium* OF09-33XD	18.8	20.5	0.0	0.0	NA	0.0482

^1^ The raw spectral counts matching to the identified bacterial species were analyzed using tweeDEseq package in Bioconductor. ^2^ HM and MF columns indicate mean value of the total spectral counts followed by the standard deviation of the mean (SD). ^3^ Log_2_ FC is the log_2_ of the HM to MF ratio. ^4^ Benjamini–Hochberg correction for multiple testing was applied to adjust *p*-values.

**Table 2:** Bacterial protein profile of cecal content of piglets (*n* = 11/group) fed either with human milk (HM) or milk formula (MF) at PND 21.

Uniprot_ID	Organism	Protein Name	HM ^1^ (sum)	MF ^1^ (sum)	HM ^2^	MF ^2^	Log_2_ FC ^3^	*p*-Value ^4^
A0A412VME7	*Phocaeicola vulgatus* (*Bacteroides vulgatus*)	RagB/SusD family nutrient uptake outer membrane protein	51	573	3.4	47.5	−3.8	0.002
A0A412VME7	*Phocaeicola dorei*	RagB/SusD family nutrient uptake outer membrane protein	51	573	3.4	45.9	−3.8	0.008
R9H865	*Bacteroides vulgatus* CL09T03C04	Uncharacterized protein	1783	111	3.4	32.9	−3.3	0.03
C6Z771	*Bacteroides* sp. 4_3_47FAA	SusD family protein	51	353	3.4	32.9	−3.3	0.03
A0A1Y3Z4G2	*Bacteroides clarus*	Polyribonucleotide nucleotidyltransferase (EC 2.7.7.8) (polynucleotide phosphorylase) (PNPase)	44	386	3.7	35.4	−3.2	0.05
A0A397WJ15	*Phocaeicola vulgatus* (*Bacteroides vulgatus*)	Beta-galactosidase (EC 3.2.1.23)	107	614	8.4	52.2	−2.6	0.02
A0A415SZ11	*Phocaeicola vulgatus* (*Bacteroides vulgatus*)	Malate dehydrogenase (EC 1.1.1.37)	1008	1997	92.3	180.3	−1	0.03
C3RBX6	*Bacteroides vulgatus* CL09T03C04	60 kDa chaperonin (GroEL protein) (protein Cpn60)	3254	1195	245.9	118.3	1.1	0.03
C3RBX6	*Bacteroides* sp. AM18-9	60 kDa chaperonin (GroEL protein) (protein Cpn60)	3254	1195	245.9	118.3	1.1	0.03
A0A069SFK4	*Bacteroides vulgatus* str. 3975 RP4	TonB-linked outer membrane, SusC/RagA family protein	1370	468	103.2	37.4	1.5	0.02
A0A069SKV9	*Bacteroides vulgatus* str. 3975 RP4	Tetracycline resistance protein TetQ	3792	1001	302.9	96.4	1.7	0.03
A0A069SKV9	*Bacteroides* sp. AF32-15BH	Tetracycline resistance protein TetQ	3792	1001	302.9	96.4	1.7	0.03
A0A1H6GDE8	*Selenomonas ruminantium*	Phosphoenolpyruvate carboxykinase (ATP) (PCK) (PEP carboxykinase) (PEPCK) (EC 4.1.1.49)	2383	519	238.1	43.5	2.5	0.04
A0A174QRN1	*Phocaeicola vulgatus* (*Bacteroides vulgatus*)	DUF1735 domain-containing protein (galactose oxidase) (EC 3.2.1.18)	171	29	13.1	2	2.7	0.03
I9UCD9	*Phocaeicola vulgatus* (*Bacteroides vulgatus*)	SusC/RagA family TonB-linked outer membrane protein	1370	469	32.3	4.7	2.8	0.03
A0A417FRM0	*Firmicutes bacterium* AM31-12AC	LacI family transcriptional regulator	251	35	24.5	2.8	3.1	0.02
A0A397WGI7	*Phocaeicola vulgatus* (*Bacteroides vulgatus*)	RagB/SusD family nutrient uptake outer membrane protein (starch-binding associating with outer membrane)	1087	111	96.8	10.5	3.2	0.02
A0A316A2W0	*Faecalicatena contorta*	Phosphoglycerate kinase (EC 2.7.2.3)	1142	96	107.1	8.7	3.6	0.002
R9H865	*Bacteroides vulgatus* dnLKV7	Uncharacterized protein	1783	111	128.5	10.5	3.6	0.01
A0A417FRM0	*Firmicutes bacterium* AM43-11BH	LacI family transcriptional regulator	251	35	21.6	1.5	3.8	0.05
R5ICG7	*Firmicutes bacterium* CAG:124	L-fucose isomerase (FucIase) (EC 5.3.1.25) (6-deoxy-L-galactose isomerase)	937	0	84.9	0	NA	<0.0001
A0A3C1BZY4	*Clostridiales bacterium*	Carbon monoxide dehydrogenase (EC 1.2.7.4)	0	535	0	53.7	NA	0.007
F0Z4P0	*Clostridium* sp. D5	Acetate kinase (EC 2.7.2.1) (acetokinase)	296	0	25.9	0	NA	0.008
A0A374BZR2	*Firmicutes bacterium* AM31-12AC	D-ribose pyranase (EC 5.4.99.62)	155	0	13.1	0	NA	0.02
A0A417BM01	*Firmicutes bacterium* AM43-11BH	UPF0210 protein DW928_02850	148	0	12.9	0	NA	0.02
A0A2N5NQW2	*Ruminococcus gnavus*	Class II fructose-1,6-bisphosphate aldolase (EC 4.1.2.13) (fructose-1,6-bisphosphate aldolase, class II)	777	0	78.2	0	NA	0.02
A0A494ZWH6	*Ruminococcus* sp. B05	UPF0210 protein D8Q48_03220	148	0	12.9	0	NA	0.02
A0A374BZR2	*Mediterraneibacter* sp. gm002	D-ribose pyranase (EC 5.4.99.62)	155	0	13.1	0	NA	0.02
A0A316A2W0	*Dorea* sp. CAG:105	Phosphoglycerate kinase (EC 2.7.2.3)	1142	96	47.3	0	NA	0.02
F0Z4P0	*Lachnospiraceae bacterium* 1_4_56FAA	Acetate kinase (EC 2.7.2.1) (acetokinase)	296	0	18.6	0	NA	0.03
A0A3A6G758	*Lachnospiraceae bacterium* TM07-2AC	Class II fructose-1,6-bisphosphate aldolase (EC 4.1.2.13)	776	0	78.1	0	NA	0.03
A0A374BZR2	*Ruminococcus* sp. AM42-11	D-ribose pyranase (EC 5.4.99.62)	155	0	12	0	NA	0.04
C3RBX6	*Firmicutes bacterium* AM43-11BH	60 kDa chaperonin (GroEL protein) (protein Cpn60)	3254	1195	107.2	0	NA	0.05

^1^ The raw spectral counts matching to the identified proteins were analyzed using tweeDEseq package in Bioconductor. ^2^ HM and MF columns indicate mean value of the total spectral counts. ^3^ Log_2_ FC is the log_2_ of the HM-to-MF ratio. ^4^ Benjamini–Hochberg correction for multiple testing was applied to adjust *p*-values.

## Data Availability

The mass spectrometry proteomics data have been deposited to the ProteomeXchange Consortium via the PRIDE partner repository with the dataset identifier PXD025432 and 10.6019/PXD025432. Reviewer login details: Username: reviewer_pxd025432@ebi.ac.uk Password: qvFTwXRs.

## Supplementary Materials

The following are available online at https://www.mdpi.com/article/10.3390/nu13113718/s1, Figure S1: Venn diagram representing the bacterial pairwise comparisons. Figure S2: Venn diagram representing the proteins pairwise comparisons. Dataset S1: All bacteria identified with the current approach. Dataset S2: All proteins identified in the experimental samples. Table S1: Host/bacteria ratios identified in this study. Table S2: Host protein expression identified in the cecal contents of piglets fed HM or MF at postnatal day 21.
