# Supplementary material for: Milk Formula Diet Alters Bacterial and Host Protein Profile in Comparison to Human Milk Diet in Neonatal Piglet Model
Source: Nutrients. 2021 Oct 22;13(11):3718. doi: 10.3390/nu13113718 (PMC8618976; doi:10.3390/nu13113718)

Intersection in DE genes − FDR < 0.05 & |logFC| > 1

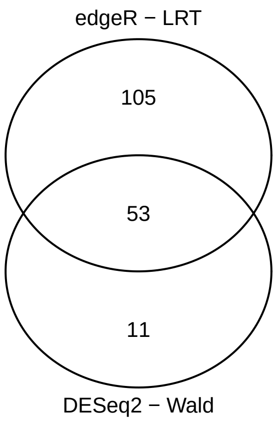

Intersection in DE genes − FDR < 0.05 & |logFC| > 1

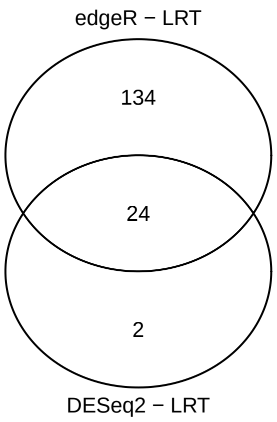

Intersection in DE genes − FDR < 0.05 & |logFC| > 1

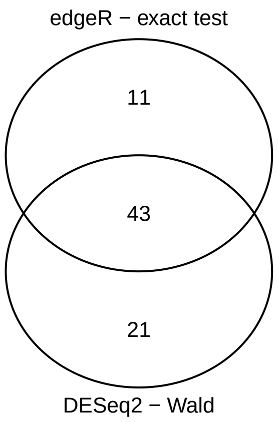

Intersection in DE genes − FDR < 0.05 & |logFC| > 1

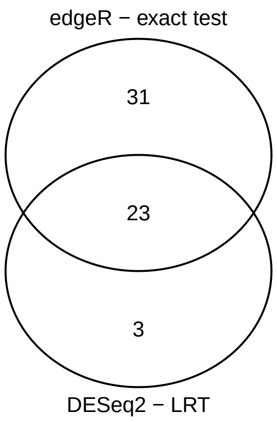

Intersection in DE genes − FDR < 0.05 & |logFC| > 1

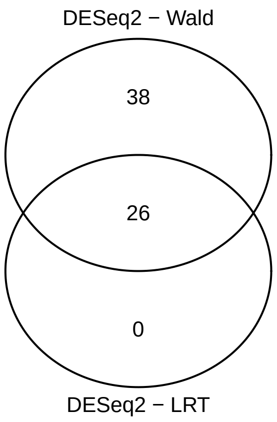

Intersection in DE genes − FDR < 0.05 & |logFC| > 1

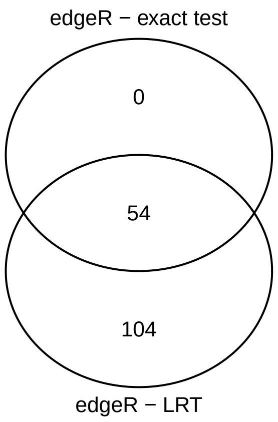

Supplement: Supplementary file 1 [file nutrients-13-03718-s001.zip › Figure S1.pdf]
