# Supplementary material for: Milk Formula Diet Alters Bacterial and Host Protein Profile in Comparison to Human Milk Diet in Neonatal Piglet Model
Source: Nutrients. 2021 Oct 22;13(11):3718. doi: 10.3390/nu13113718 (PMC8618976; doi:10.3390/nu13113718)
Supplement: Supplementary file 1 [file nutrients-13-03718-s001.zip › Figure S2.pdf]

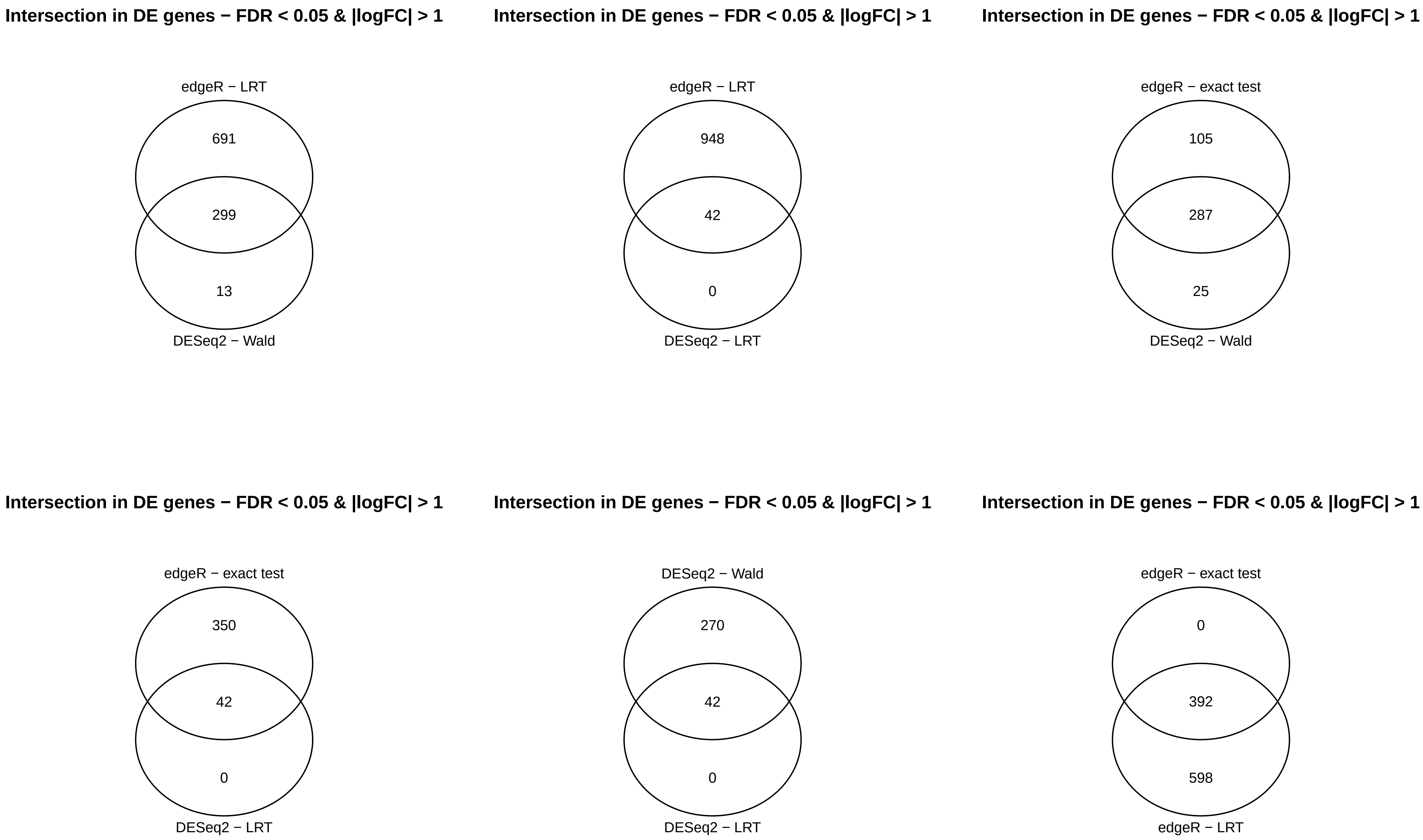
Intersection in DE genes − FDR < 0.05 & |logFC| > 1
edgeR − LRT
691
299
13
DESeq2 − Wald
Intersection in DE genes − FDR < 0.05 & |logFC| > 1
edgeR − LRT
948
42
0
DESeq2 − LRT
Intersection in DE genes − FDR < 0.05 & |logFC| > 1
edgeR − exact test
105
287
25
DESeq2 − Wald
Intersection in DE genes − FDR < 0.05 & |logFC| > 1
edgeR − exact test
350
42
0
DESeq2 − LRT
Intersection in DE genes − FDR < 0.05 & |logFC| > 1
DESeq2 − Wald
270
42
0
DESeq2 − LRT
Intersection in DE genes − FDR < 0.05 & |logFC| > 1
edgeR − exact test
0
392
598
edgeR − LRT
