# Supplementary material for: Milk Formula Diet Alters Bacterial and Host Protein Profile in Comparison to Human Milk Diet in Neonatal Piglet Model
Source: Nutrients. 2021 Oct 22;13(11):3718. doi: 10.3390/nu13113718 (PMC8618976; doi:10.3390/nu13113718)
Supplement: Supplementary file 1 [file nutrients-13-03718-s001.zip › Supplemental Table 1.pdf]

**Supplemental Table 1**. Host/Bacteria ratios identified in this study.

| Total MS/MS spectra | Human milk | | | | | | | | | | | |
|---|---|---|---|---|---|---|---|---|---|---|---|---|
| | animal 35 | animal 36 | animal 37 | animal 67 | animal 68 | animal 71 | animal 72 | animal 75 | animal 76 | animal 82 | animal 83 | Total |
| Homo sapiens (Human) | 24378 | 19869 | 13388 | 50818 | 38721 | 58320 | 31624 | 37409 | 54093 | 31511 | 58316 | 388 |
| Sus Scrofa (Pig) | 45245 | 49001 | 61923 | 80214 | 111565 | 125831 | 56245 | 142106 | 199335 | 73029 | 99911 | 900 |
| Bacteria | 96751 | 172371 | 160738 | 93744 | 221952 | 308011 | 223612 | 170022 | 172546 | 116825 | 80674 | 7396 |
| %Bacteria | 58.15 | 71.45 | 68.10 | 41.71 | 59.63 | 62.58 | 71.79 | 48.64 | 40.51 | 52.77 | 33.77 | 85.17 |
| | Milk formula | | | | | | | | | | | |
| | animal 38 | animal 39 | animal 40 | animal 69 | animal 70 | animal 73 | animal 74 | animal 77 | animal 79 | animal 80 | animal 81 | |
| Homo sapiens (Human) | 10818 | 54148 | 32724 | 30176 | 22863 | 37187 | 20804 | 51120 | 37028 | 30793 | 22930 | |
| Sus Scrofa (Pig) | 41192 | 81455 | 65460 | 124713 | 108086 | 111259 | 117072 | 182276 | 153121 | 140549 | 63466 | |
| Bacteria | 47114 | 141495 | 177483 | 203312 | 142029 | 122392 | 130915 | 132025 | 179625 | 190252 | 123124 | |
| %Bacteria | 47.53 | 51.06 | 64.38 | 56.76 | 52.03 | 45.19 | 48.71 | 36.13 | 48.58 | 52.61 | 58.76 | |
| | Human milk | | | | | | | | | | | |
| Proteins ID-ed | animal 35 | animal 36 | animal 37 | animal 67 | animal 68 | animal 71 | animal 72 | animal 75 | animal 76 | animal 82 | animal 83 | |
| Homo sapiens (Human) | 154 | 83 | 121 | 172 | 205 | 240 | 142 | 220 | 259 | 190 | 257 | |
| Sus Scrofa (Pig) | 534 | 382 | 516 | 487 | 532 | 469 | 352 | 519 | 644 | 437 | 558 | |
| Bacteria | 3225 | 2785 | 2998 | 2164 | 3524 | 3164 | 3220 | 3134 | 3730 | 3116 | 2930 | |
| %Bacteria | 82.42 | 85.69 | 82.48 | 76.66 | 82.70 | 81.69 | 86.70 | 80.92 | 80.51 | 83.25 | 78.24 | |
| | Milk formula | | | | | | | | | | | |
| | animal 38 | animal 39 | animal 40 | animal 69 | animal 70 | animal 73 | animal 74 | animal 77 | animal 79 | animal 80 | animal 81 | |
| Homo sapiens (Human) | 95 | 123 | 107 | 95 | 93 | 161 | 107 | 168 | 149 | 164 | 105 | |
| Sus Scrofa (Pig) | 463 | 446 | 416 | 507 | 522 | 559 | 626 | 603 | 531 | 490 | 445 | |
| Bacteria | 2633 | 3255 | 2824 | 3595 | 3322 | 2730 | 3658 | 3305 | 3398 | 3178 | 2565 | |
| %Bacteria | 82.51 | 85.12 | 84.37 | 85.66 | 84.38 | 79.13 | 83.31 | 81.08 | 83.33 | 82.93 | 82.34 | |
