# Supplementary material for: Milk Formula Diet Alters Bacterial and Host Protein Profile in Comparison to Human Milk Diet in Neonatal Piglet Model
Source: Nutrients. 2021 Oct 22;13(11):3718. doi: 10.3390/nu13113718 (PMC8618976; doi:10.3390/nu13113718)
Supplement: Supplementary file 1 [file nutrients-13-03718-s001.zip › Supplemental Table 2.pdf]

Supplemental Table 2. Host protein expression in piglets fed either with human milk (HM) or milk formula (MF) at postnatal day 21.

Organism[1]	Protein name	HM[2]	MF[2]	log2 FC[3]	*P*-value[4]
Pig	Secreted folate binding protein	6.5	89.8	-3.8	0.03
Pig	Folate_rec domain-containing protein	6.6	98.5	-3.9	0.002
Pig	Transthyretin	24.4	172.5	-2.8	0.008
Human	N-sulphoglucosamine sulphohydrolase (EC 3.10.1.1) (Sulfoglucosamine sulfamidase) (Sulphamidase)	17.9	0.3	6.0	0.03
Human	Lactotransferrin	938.3	16.2	5.9	0.03
Human	Alpha-1-antitrypsin (Serpin peptidase inhibitor, clade A (Alpha-1 antiproteinase, antitrypsin), member 1, isoform CRA_a)	64.3	1.6	5.3	0.01
Human	Epididymis secretory sperm binding protein Li 44a (Serpin peptidase inhibitor clade A member 1 isoform 1)	63.2	1.6	5.3	0.03
Human	N(4)-(beta-N-acetylglucosaminyl)-L-asparaginase (EC 3.5.1.26) (Aspartylglucosaminidase) (Glycosylasparaginase) (N4-(N-acetyl-beta-glucosaminyl)-L-asparagine amidase) [Cleaved into: Glycosylasparaginase alpha chain; Glycosylasparaginase beta chain]	57.0	6.0	3.2	0.01
Human	Ubiquitin C	31.3	4.7	2.7	0.02
Human	Ferritin	91.5	0.0	NA	0.02
Human	cDNA FLJ35730 fis, clone TESTI2003131, highly similar to ALPHA-1-ANTICHYMOTRYPSIN	39.3	0.0	NA	0.02
Human	Galectin-3-binding protein (Basement membrane autoantigen p105) (Lectin galactoside-binding soluble 3-binding protein) (Mac-2-binding protein) (MAC2BP) (Mac-2 BP) (Tumor-associated antigen 90K)	57.6	0.0	NA	0.02
Human	Alpha-1-antichymotrypsin (ACT) (Cell growth-inhibiting gene 24/25 protein) (Serpin A3) [Cleaved into: Alpha-1-antichymotrypsin His-Pro-less]	52.7	0.0	NA	<0.0001
Human	Serpin peptidase inhibitor, clade A (Alpha-1 antiproteinase, antitrypsin), member 3, isoform CRA_c	52.7	0.0	NA	<0.0001

[1]The raw spectral counts matching to the identified proteins were analyzed using tweeDEseq package in Bioconductor. ^{2}HM and MF (n = 11/group) columns indicated mean value of the total spectral counts. [3]Log2 FC is the log2 of the HM to MF ratio. [4]Benjamini-Hochberg correction for multiple testing was applied to adjust *P*-values.
